# Single-cell transcriptomic profiling unveils insights into ovarian fibrosis in obese mice

**DOI:** 10.1186/s13062-024-00496-9

**Published:** 2024-07-02

**Authors:** Bang Xiao, Zhihui Dai, Zhixuan Li, Dabing Xu, Haozan Yin, Fu Yang, Ningxia Sun

**Affiliations:** 1 Department of Medical Genetics, Naval Medical University, 800 Xiang yin Road, Shanghai, 200433 China; 2 Department of Reproductive Medicine, Second Affiliated Hospital of Naval Medical University, 415 Feng yang Road, Shanghai, 200003 China; 3 Translational Medicine Research Center, Medical Innovation Research Division and Fourth Medical Center of the Chinese PLA General Hospital, 51 Fu cheng Road, Beijing, 100853 China

**Keywords:** Single-cell sequencing, Obesity, Fibrosis, Granulosa cell, SPP1

## Abstract

**Background:**

Adiposity profoundly impacts reproductive health in both humans and animals. However, the precise subpopulations contributing to infertility under obese conditions remain elusive.

**Results:**

In this study, we established an obese mouse model through an eighteen-week high-fat diet regimen in adult female mice. Employing single-cell RNA sequencing (scRNA-seq), we constructed a comprehensive single-cell atlas of ovarian tissues from these mice to scrutinize the impact of obesity on the ovarian microenvironment. ScRNA-seq revealed notable alterations in the microenvironment of ovarian tissues in obese mice. Granulosa cells, stromal cells, T cells, and macrophages exhibited functional imbalances compared to the control group. We observed heightened interaction strength in the SPP1-CD44 pairing within lgfbp7^+^ granulosa cell subtypes and Il1b^high^ monocyte subtypes in the ovarian tissues of obese mice. Moreover, the interaction strength between Il1b^high^ monocyte subtypes and Pdgfrb^+^ stromal cell subtypes in the form of TNF − TNFrsf1α interaction was also enhanced subsequently to obesity, potentially contributing to ovarian fibrosis pathogenesis.

**Conclusions:**

We propose a model wherein granulosa cells secrete SPP1 to activate monocytes, subsequently triggering TNF-α secretion by monocytes, thereby activating stromal cells and ultimately leading to the development of ovarian fibrosis. Intervening in this process may represent a promising avenue for improving clinical outcomes in fertility treatments for obese women.

**Supplementary Information:**

The online version contains supplementary material available at 10.1186/s13062-024-00496-9.

## Background

More than two-thirds of women of reproductive age are afflicted by overweight or obesity (OB), characterized by a body mass index (BMI) exceeding 25 kg/m²^2^. This condition predisposes them to heightened risks of infertility and pregnancy-related complications, necessitating increased utilization of fertility treatments [1]. When compared to women within the normal-weight (NW) range, OB individuals exhibit substantially reduced yields of retrieved oocytes, diminished quantities of cleaved embryos, decreased production of high-grade embryos, and elevated rates of miscarriage in the context of in vitro fertilization (IVF) procedures [2]. Furthermore, research has demonstrated a decline in pregnancy and live birth rates correlated with higher BMIs among oocyte donors, irrespective of the recipients’ BMI [3]. Notably, OB women have been found to display significantly elevated levels of the serum homeostatic model assessment-insulin resistance index in contrast to their NW counterparts [4]. Additionally, oocytes derived from OB women exhibit heightened expression of proinflammatory and oxidative stress-related genes relative to those from NW women [5].

Oocytes, integral to the reproductive process, are maintained in a state of meiotic arrest within the ovarian follicles, ensconced amidst extracellular matrix and embedded within stromal interstitial tissue [6]. Recent investigations have brought to light increased collagen deposition in the ovaries of obese women and animal models of obesity, indicative of tissue fibrosis [7, 8]. Umehara has elucidated that obesity-induced ovarian fibrosis bears resemblance to the fibrosis observed in reproductive aging, attributing mitochondrial damage in stromal cells as the pivotal instigating factor for inflammation and fibrosis-induced ovarian decline [9]. Nevertheless, the precise cellular events driving ovarian fibrosis in the context of obesity remain inadequately defined. It is plausible that accumulated collagen hampers follicular growth and obstructs ovulation, effectively precipitating premature ovarian failure by impeding the release of mature, fertilization-competent oocytes. A thorough comprehension of the molecular alterations occurring in the ovaries of OB women and animal models before conception may furnish valuable insights into the etiology of infertility, thereby paving the way for novel intervention strategies aimed at enhancing female fertility.

The ovarian milieu is characterized by a diversity of cell types, states, and intricate intercellular interactions [10]. Moreover, gene expression profiles vary substantially across distinct cell types and under differing conditions within the ovaries, making it challenging to discern such nuanced distinctions when employing conventional research approaches centered on whole ovaries or follicles [11]. This limitation arises because bulk RNA sequencing merely permits comparisons of average gene expression, thereby precluding a comprehensive exploration of cellular-level discrepancies. The advent of scRNA-seq technology has revolutionized the capacity to detect variations in transcriptome expression among specific ovarian cell types, facilitating the delineation of cellular development pathways and the elucidation of intercellular communication networks [12, 13]. Presently, scRNA-seq has been effectively employed to construct a developmental map of germ cells in the mouse ovaries during meiotic initiation and primordial follicle assembly [14, 15]. Furthermore, Wang et al. employed scRNA-seq to contrast transcriptional distinctions at the single-cell level between young and aged ovaries in non-human primates, offering valuable insights into the molecular underpinnings of ovarian aging [14]. This underscores the utility of scRNA-seq in investigating how adiposity affects ovarian cells at the molecular level.

In the current study, we harnessed single-cell RNA-seq to construct a comprehensive single-cell map of ovarian tissues in obese and normal-weight mice. Our analysis of intercellular communication unveils intricate relationships among diverse cell types, highlighting granulosa cells and ovarian stromal cells as the principal cell types engaged in outgoing signaling, while monocytes emerge as the predominant cell type exerting incoming interaction strength in the ovarian microenvironment. These findings underscore the substantial impact of adiposity on ovarian fibrosis and offer a promising avenue for addressing reproductive health challenges stemming from obesity.

## Methods

### Animals subjects

The C57BL/6j mice utilized in this investigation were procured from SLAC Laboratory Animal Co., Ltd. (Shanghai, China). The animals were managed in accordance with prevailing regulations [GB14925-2001: Laboratory Animal Requirements of Environment and Housing Facilities (Chinese version)] and received humane care. All animal treatments and experimental protocols in this study were sanctioned by the Institutional Ethics Committee of the Naval Medical University (approval no. 20,190,022).

### Human participants

Healthy women, aged 21 to 38 years, undergoing fertility treatment involving oocyte retrieval with an equal ovarian stimulation procedure for everybody which didn’t affect the results SPP1 level evaluation at the Center of Reproductive Medicine, Shanghai Changzheng Hospital, were recruited based on their BMI: normal weight (18.5 to 24.9 kg/m^2^; *N* = 17) and obesity (≥ 25 kg/m^2^; *N* = 12). Participants’ infertility diagnoses included 20% male factor, 15% endometriosis, 25% ovulatory disorder, 20% tubal factor, and 20% unexplained (see Supplementary Table S1). Women with diagnoses of polycystic ovary syndrome, cardiovascular diseases, or diabetes were excluded. The study protocol was approved by the Shanghai Changzheng Hospital Medical Ethics Committee, and informed consent was obtained from all participants.

### Establishment of the obese female mouse model

Female C57BL/6j mice were randomly assigned to experimental and control groups. Mice in the experimental group were fed a high-fat diet comprising 60 kcal% fat, while those in the control group received a standard diet. Both groups had ad libitum access to food, and their body weights were monitored weekly. The obese mouse model was confirmed when the body weight of the experimental group mice exceeded that of the control group by 20%^38^.

### Single-cell RNA-seq library preparation and sequencing

Ovaries were collected from individual mice, with 10 ovaries obtained from each group to ensure an adequate number of cells and repeatability. Ovaries were minced and enzymatically digested using 0.25% trypsin (Sangon Biotech, Shanghai, China, A003702) and collagenase (2 mg/ml, Sigma, C5138) at 37 °C for 6–8 min. The resulting cell suspension was filtered through 40 μm cell filters (BD Falcon, USA, 352,340) and washed thrice with 0.04% bovine serum albumin (BSA, Sigma, A1933) in PBS. Cell concentration and viability were assessed using Trypan blue and a Countess Automated Cell Counter (Thermo Fisher Scientific, Waltham, USA). Cells meeting specified viability criteria were used for subsequent analyses.

Single-cell RNA-Seq libraries were constructed using the SeekOne MM Single Cell 3’ Library Preparation Kit (SeekGene, Catalog No. K00104-04). Briefly, an appropriate number of cells were loaded into the SeekOne MM chip containing 170,000 microwells. Cells were allowed to settle in the microwells by gravity, and non-settled cells were removed. Cell barcoded magnetic beads (CBBs) were introduced into the chip and allowed to settle in the microwells under a magnetic field. Excess CBBs were washed out, and cell lysis was performed to release captured RNA. Reverse transcription labeled the cDNA with cell barcodes on the beads, followed by exonuclease I treatment to remove unused primers on the CBBs. The barcoded cDNA on CBBs was hybridized with random primers, forming the second-strand DNA with cell barcodes. The resulting DNA was denatured off the CBBs, purified, and amplified by polymerase chain reaction (PCR). The amplified cDNA product was cleaned, adapter-ligated, and indexed before sequencing on an Illumina NovaSeq 6000 machine with PE150 read length.

### Processing of single-cell RNA sequencing data

Raw sequencing data underwent initial processing with Fastp to trim primer sequences and low-quality bases. SeekOneTools (Beijing SeekGeneBioSciences) was employed for subsequent data processing. Seurat (version 4.0.3) was used to filter out low-quality cells and cells with a mitochondrial gene percentage between 20% and 40%, retaining cells with a detected gene count between 200 and 6000 genes.

### Dimensionality reduction and clustering analysis

Following quality control and filtering, Seurat (V4.0.3) was employed for clustering and visualization. Data normalization was performed for each cell, variable genes were identified, and libraries were integrated using default parameters. Dimensionality reduction and cell clustering were performed, and cluster-specific marker genes were identified. Differentially expressed genes (DEGs) were considered significant if the adjusted P value was < 0.05 and the avg._log2FC was greater than or equal to 0.25.

### Cell-cell interactions

CellPhoneDB was utilized to infer cell-cell interactions, utilizing log-transformed and normalized counts along with cell-type annotations as inputs. Specific interactions were identified based on ligand and receptor expression in more than 10% of cells within a cluster.

### Pseudotime analysis

Monocle software (version 2.3.6) was employed to investigate potential lineage differentiation among cellular populations. CellDataSet objects were created, and genes used for pseudotime ordering were determined using the dispersion Table function. Dimension reduction and cell ordering were performed using the DDRTree method, followed by branch analysis. Genes significantly changed at the branch point were clustered based on distinct patterns of gene-expression changes.

### Enrichment analysis

Differentially expressed genes were selected based on P-value, and Gene Ontology (GO) and Kyoto Encyclopedia of Genes and Genome (KEGG) analyses were conducted using the R Bioconductor/cluster Profiler package. GO terms or KEGG pathways with a corrected P value < 0.05 were considered significantly enriched by marker genes.

### Ovary immunostaining

Intact ovaries were fixed in 4% paraformaldehyde (PFA) at 4 °C overnight, processed through standard histological embedding procedures, and sectioned into 5 μm thick slices. After deparaffinization and rehydration, slides were subjected to antigen retrieval and blocking with BDT (3% BSA, 10% normal goat serum in TBS). Primary and secondary antibodies were applied, followed by nuclear staining with Hoechst 33,342 (Beyotime, C1022) or PI (Solarbio, P8080, Beijing, China).

Immunohistochemistry followed the same procedure as described above, with the addition of 3% H_2_O_2_ treatment before blocking. Standard wells were set up for measurement at 450 nm wavelength.

### Reverse transcription quantitative polymerase chain reaction (RT-qPCR)

Total RNA was extracted from ovarian tissue using RNAiso Plus (Takara, Shiga, Japan) according to the manufacturer’s instructions. The Hifair II 1st Strand cDNA Synthesis Kit (Yeasen Biotechnology, Shanghai, China) was used for cDNA synthesis, followed by qPCR using 2 × ChamQ Universal SYBR qPCR Master Mix (Vazyme Biotech, Nanjing, China) and specific primers. β-actin served as the reference control, and data analysis utilized the 2^^−ΔΔct^ method. The sequences of all the primers used were listed in Supplementary Table S2.

### Western blotting

Cells collected at predetermined time points were lysed using RIPA lysate solution for protein extraction. Protein samples were subjected to SDS-PAGE gel electrophoresis, transferred to a PVDF membrane, and blocked with 5% BSA. Primary and secondary antibodies were applied, and protein bands were visualized using chemiluminescence.

### Enzyme-linked immunosorbent assay (ELISA)

Whole blood was collected from mice via the orbital venous plexus for serum isolation and determination of serum levels of anti-Müllerian hormone (AMH), follicle-stimulating hormone (FSH), and estradiol (E2). Follicular fluid and granulosa cells from patients undergoing fertility treatment were also subjected to ELISA to measure secreted phosphoprotein 1 (SPP1) levels.

### Statistical analysis

Data were obtained from at least three independent replicates and are presented as mean ± SD. Statistical significance was assessed using GraphPad Prism 8.0 software, employing the t-test or one-way analysis of variance (ANOVA) as appropriate.

## Results

### The impact of obesity on ovarian function in mice

**Fig. 1:**
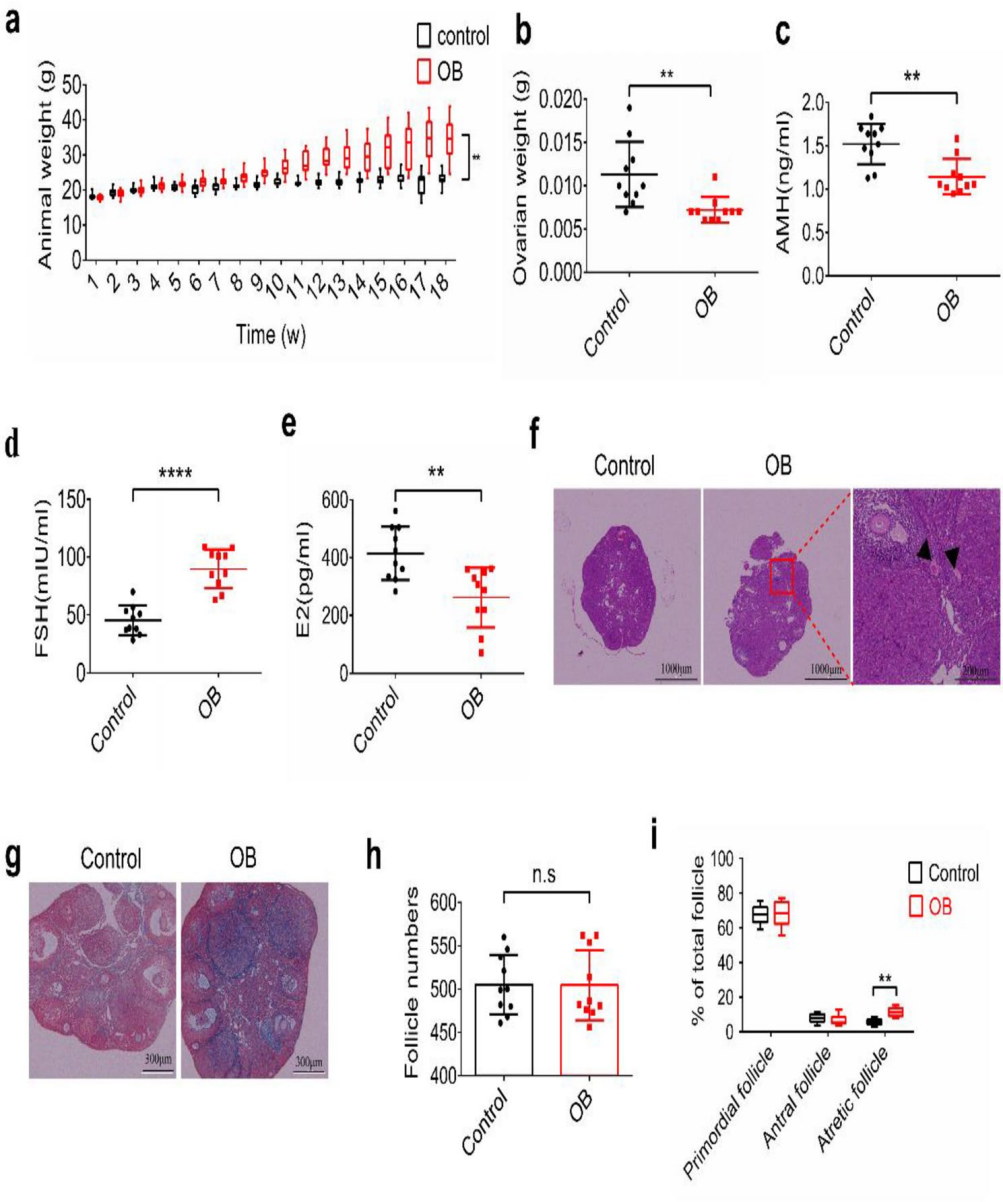
Obesity reduces ovarian function in mice. **a** High-fat diet (HFD) was administered daily to adult female mice for eighteen weeks to construct the obese mouse model and the weight of mice were measured. **b** The mice were euthanized and the ovarian weight of two groups were measured after HFD treatment for eighteen weeks. **c-e** The levels of anti-Müllerian hormone (AMH), follicle-stimulating hormone (FSH) and estradiol (E2) in serum of two groups of mice were measured by ELISA. **f** Hematoxylin-eosin (HE) staining was used to observe the pathological changes of ovarian tissue. **g** Masoon staining was used to observe the degree of ovarian fibrosis in two groups of mice. **h** Total number of follicles between the OB group and the normal (N) group were counted. **i** The proportion of atretic follicles in two groups were calculated. All experiments were repeated at least three times (n.s, not significant; *P < 0.05; **P < 0.01; ***P < 0.001; ****P < 0.0001)

To investigate the repercussions of obesity on ovarian function, we administered a high-fat diet (HFD) to adult female mice daily for eighteen weeks to establish an obese mouse model. Subsequently, we assessed ovarian function. Our results from weight measurements revealed a significant increase in the weight of mice subjected to HFD treatment compared to the control groups (Fig. 1a). However, the ovarian weight in the obese group (OB) was notably lower than that in the control group (Fig. 1b). We employed ELISA to measure the levels of AMH, FSH, and E2 in the serum of both groups of mice. Our findings indicated that, in the OB group, the levels of AMH and E2 were significantly reduced, whereas FSH levels were significantly elevated compared to the control group (Fig. 1c-e). Hematoxylin-eosin (HE) staining was utilized to assess pathological changes in ovarian tissue (Fig. 1f). The results showed no significant difference in the total number of follicles between the OB group and the control group. However, the proportion of atretic follicles in the OB group was significantly higher than that in the control group (Fig. 1h, i). Masoon staining revealed a higher degree of ovarian fibrosis in the OB group compared to the control group (Fig. 1g). Collectively, these data suggest that obesity leads to a substantial reduction in ovarian function in female mice.

### Identification of cell population types within mouse ovaries in OB and normal groups

**Fig. 2:**
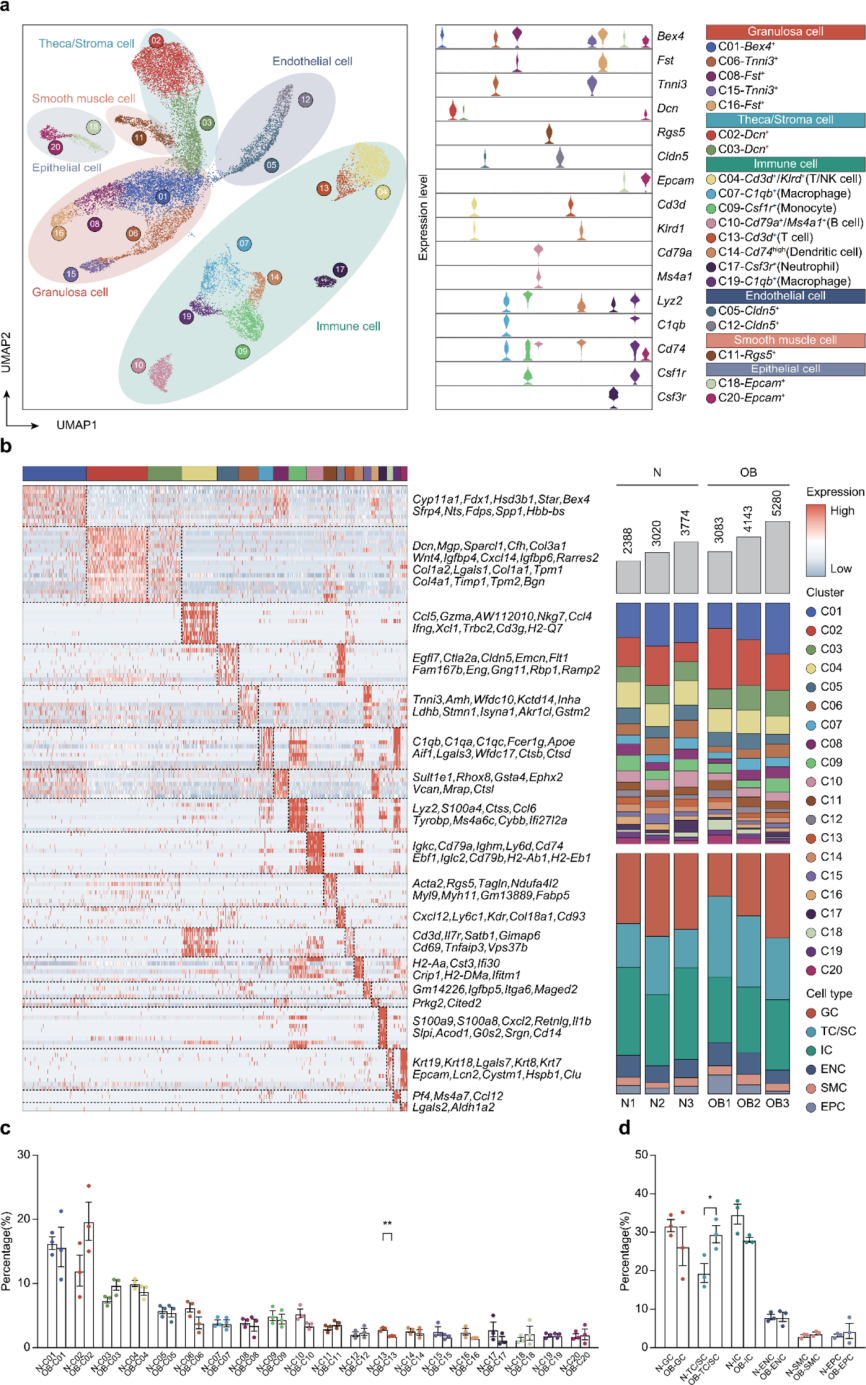
Single-cell sequencing results and identification of cell cluster types in ovaries. **a** Annotated UMAP of 21,688 high-quality cells grouped into 20 semisupervised clusters and labeled according to cell type and violin charts of identifying marker genes. **b** Heatmap of genes specifically expressed in different cell clusters and fraction of cells in each cell type per sample. **c** The change in the percentage of each cell cluster after HFD treatment. **d** The change in the percentage of each cell type after HFD treatment. (*P < 0.05; **P < 0.01)

We prepared single-cell suspensions from the ovaries of two groups of mice and performed scRNA-seq. We filtered out low-quality cells based on gene count and expression levels, resulting in 21,688 ovarian cells, with 9,182 cells from the normal groups and 12,506 cells from the OB groups (Supplementary Table S3). We integrated the data from all six samples and generated 20 cell clusters using Uniform Manifold Approximation and Projection for Dimension Reduction (UMAP) (Fig. 2a). To better distinguish and classify ovarian cell populations, we employed violin plots to visualize the expression of specific marker genes in different cell clusters, ultimately categorizing ovarian cells into six distinct cell types (Fig. 2a). We further identified and displayed genes specifically expressed in different cell clusters using a heatmap (Fig. 2b). Additionally, we analyzed the differences in the percentage of each cell cluster between the normal group and OB group. Notably, there was a significant decrease in the percentage of cell cluster 13 after HFD treatment (Fig. 2b, c). As shown in Fig. 2a, cell cluster 13 corresponds to immune cells, leading us to speculate that obesity induces alterations in the immune microenvironment within ovarian tissue. Importantly, we also observed an increase in the percentage of theca/stroma cell or smooth muscle cell types in the OB group (Fig. 2d). This may play a pivotal role in the process of ovarian fibrosis.

### High-resolution analysis of obesity’s effects on stromal and theca cells

**Fig. 3:**
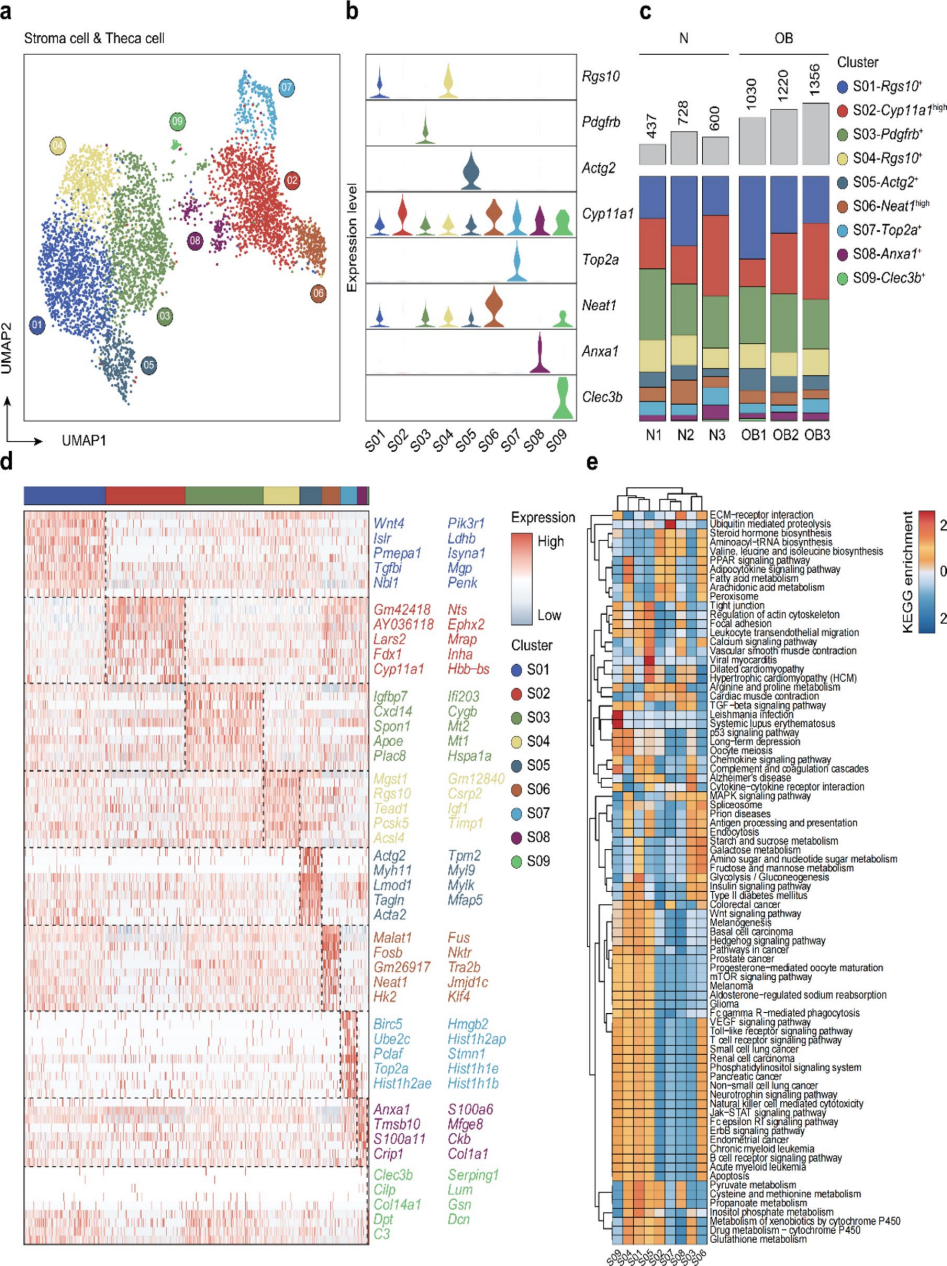
High-resolution analysis of the effect of obesity on stroma cell & theca cell. **a** Extracted stroma cell & theca cell clusters from all ovarian cells. **b** Violin charts of identifying marker genes of stroma cell subsets. **c** Fraction of stroma cells in each cell cluster per sample. **d** Heatmap of genes differentially expressed in different stroma cell subsets. **e** KEGG-pathway terms and enriched for each stroma cell cluster

Stromal cells play a critical role in follicle development, as they maintain contact with developing follicles and provide local biochemical signals for follicle growth. Following our observations of obesity’s impact on granulosa cells, we examined its effects on stromal cells. UMAP analysis of stromal cells revealed nine cell clusters encompassing stromal and theca cells (S01-S09) (Fig. 3a). Violin plots were used to visualize marker gene expression in each cell cluster (Fig. 3b). Upon analyzing the proportion of each cell subpopulation, we found no significant differences in the subpopulation proportions of stromal cells between the OB and normal groups (Fig. 3c, Supplementary Table S3). We identified DEGs between the OB and normal groups within different subpopulations, revealing that most DEGs differed among the nine sets (Fig. 3d, Supplementary Table S3). This suggests that obesity influences stromal cells in various subpopulations by affecting numerous genes. Through KEGG pathway enrichment analysis of gene sets in different groups, we noted significant enrichment in “ECM − receptor interaction,” “Ubiquitin-mediated proteolysis,” “Steroid hormone biosynthesis,” and “Valine, leucine, and isoleucine biosynthesis” in the nine groups.

### Characterization of immune cells

**Fig. 4:**
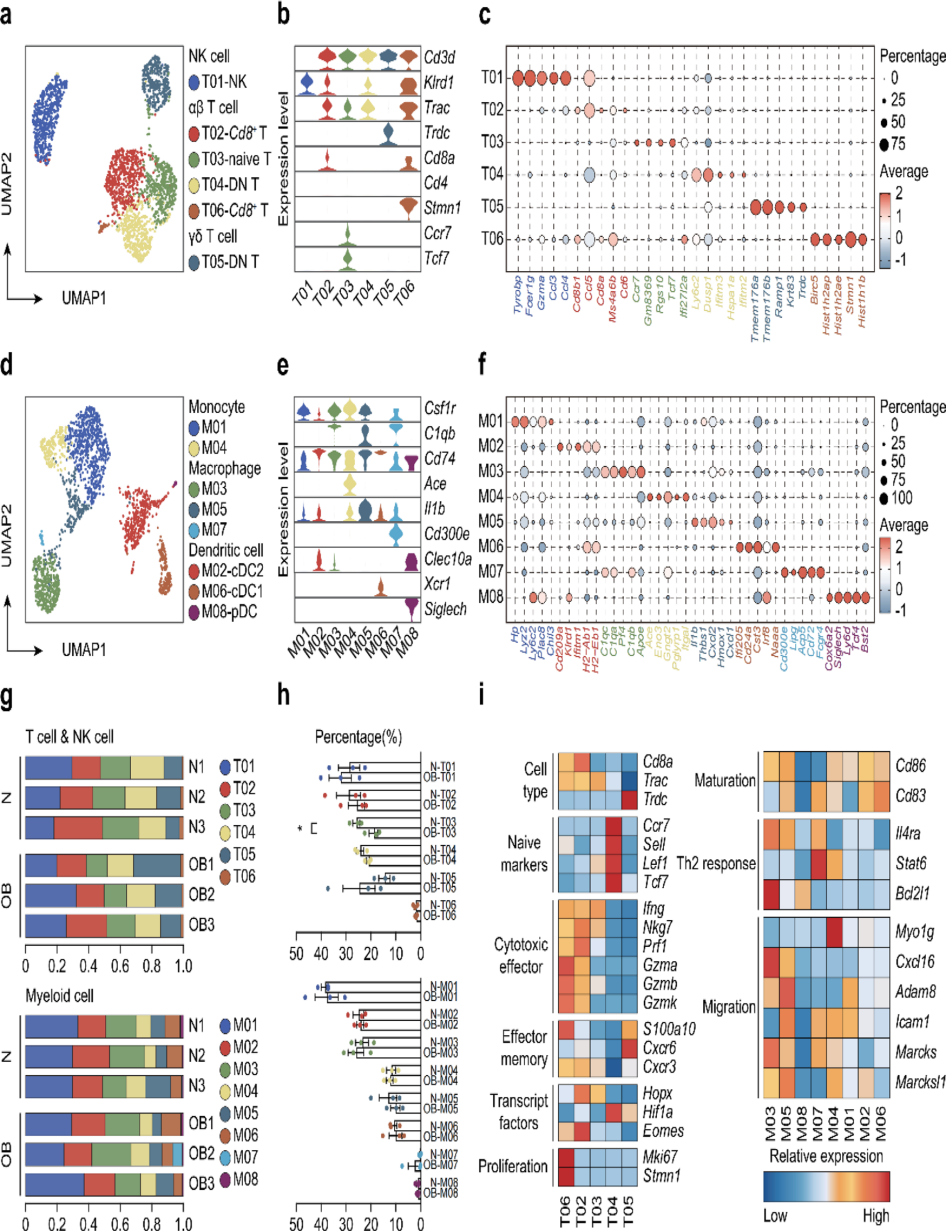
Characterization of immune cells. **a** Extracted T cell & NK cell clusters from all ovarian cells. **b** Violin plots showing expression of one representative differential expressed gene for each T cell & NK cell cluster. **c** Dot plot of five identifying marker genes for each T cell & NK cell cluster. **d** Extracted myeloid cell clusters from all ovarian cells. **e** Violin plots showing expression of one representative differential expressed gene for each myeloid cell cluster. **f** Dot plot of five identifying marker genes for each myeloid cell cluster. **g** Fraction of T cell & NK cell (upper part) and myeloid cell (lower part) in each cell cluster per sample. **h** Proportion of each T cell & NK cell cluster (upper part) and myeloid cell cluster (lower part) in all cells after HFD treatment. **i** Differentially expressed immune marker genes and enrichment in all T cell subtypes (left part) and myeloid cell (right part). (*P < 0.05; **P < 0.01)

Given the pivotal role of inflammatory cell infiltration in ovarian fibrosis development, we investigated how obesity influenced the composition of inflammatory cells in ovarian tissue. By analyzing the expression of specific marker genes, we subdivided NK cells and T cells into six cell subsets (T01-T06) (Fig. 4a, b). Additionally, we employed dot plots to visualize average expression (dot color) and percent expression (dot size) of markers in different cell populations (Fig. 4c, Supplementary Table S3). We also analyzed monocytes, macrophages, and dendritic cells, dividing them into eight subpopulations (M01-M08) (Fig. 4d, e). Key genes within these eight cell subpopulations were identified (Fig. 4f, Supplementary Table S3). Upon examining changes in the proportion of individual cell subsets between the OB group and normal group, we observed a significant decrease in the percentage of naive T cell subpopulations in the OB group (Fig. 4g, h, Supplementary Table S3). This suggests a reduction in subsequent antigen-specific and memory T cells, potentially weakening immunity against existing antigens and affecting the immune microenvironment balance in ovarian tissue. Furthermore, we identified DEGs in different cell subpopulations, noting relatively high expression of cell type-related genes such as Trdc in the T05 subpopulation, naive markers Ccr7, Sell, Lef1, and Tcf7 in the T04 subpopulation, and proliferation genes Mki67 and Stmn1 in the T06 subpopulation (Fig. 4i, Supplementary Table S3). Additionally, we found relatively high expression of the Th2 response-related gene Bcl2l1 in the M03 subpopulation and migration-related gene Myo1g in the M04 subpopulation. All these results suggest that obesity disrupts the function of immune cells within the ovaries.

### Unraveling changes in cell fates of granulosa cells impacted by obesity

**Fig. 5:**
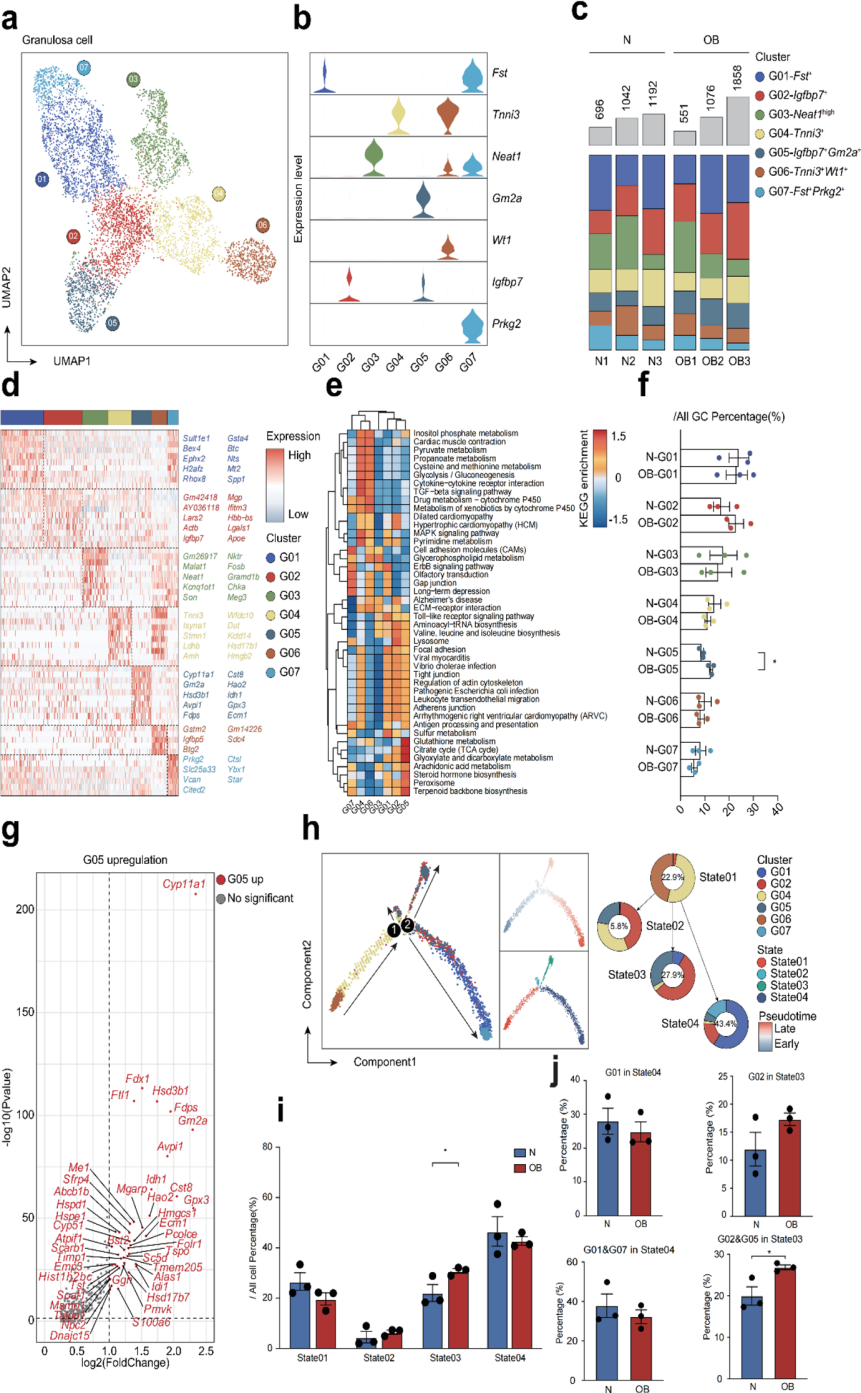
Analysis of the heterogeneity of granulosa cell subsets. **a** Extracted granulosa cell clusters from all ovarian cells. **b** Violin charts of identifying marker genes of granulosa cell subsets. **c** Fraction of granulosa cells in each cell cluster per sample. **d** Heatmap of genes differentially expressed in different granulosa cell subsets. **e** KEGG-pathway terms and enriched for each granulosa cell cluster. **f** Proportion of each granulosa cell cluster in all granulosa cells after HFD treatment. **g** The upregulated genes in G05 cell subpopulation after HFD treatment were showed. **h** Single-cell pseudotime developmental trajectory of granulosa cells, which are colored according to cell development state. **i** Proportion of the four cell states of the granulosa cells in OB group and normal group. **j** Proportion of G01or G01&G07 subpopulation in state4 and G02 or G02&G05 in state3. (*P < 0.05; **P < 0.01)

To analyze the heterogeneity of granulosa cells at various stages following HFD treatment, we extracted granulosa cell clusters for subpopulation analysis. UMAP analysis revealed the division of granulosa cells into seven clusters (G01-G07) (Fig. 5a). We identified genes specifically expressed in these seven cell clusters (Fig. 5b). Subsequently, we analyzed the percentage of each subpopulation among all granulosa cells. We observed a significant increase in the percentage of the G05 subpopulation in the OB group compared to the normal group (Fig. 5c, f). Next, we presented a heatmap displaying differentially expressed genes (DEGs) and KEGG enrichment among the seven cell clusters between the OB and normal group (Fig. 5d, e). Notably, we focused on the upregulated genes in the G05 subpopulation. We observed significant upregulation of genes such as Cyp11a1, Fdx1, Flt1, Gm2a, and S100a6 in the G05 subpopulation under the influence of obesity (Fig. 5g, Supplementary Fig. S1a, b). Moreover, we also observed the expression of Cyp11a1, which was the most significantly upregulated in G05 subpopulation in all cell clusters or six cell types (Supplementary Fig. S1c). Cyp11a1 encoding the cholesterol side chain lyase catalyzes the synthesis of pregnenolone, a common precursor of all steroid hormones, and is essential for the synthesis of vertebrate steroid hormones. Gene expression is regulated by transcriptional factors. We identified 23 transcription factors in the G05 subpopulation that may be involved in regulating gene expression (Supplementary Fig. S1d).

To elucidate the effects of obesity on granulosa cells, we investigated the pseudotime series of all granulosa cells. We observed three branches in the overall granulosa cell lineage trajectory, which determined three distinct cell fates (Fig. 5h). Granulosa cells in state 1 belonged to the pre-branch of the developmental trajectory. States 3 and 4 represented the two primary cell fates in granulosa cell development. Interestingly, we found that obesity significantly increased the percentage of granulosa cells in state 3 (Fig. 5i). Considering that state 3 predominantly comprised the G02 and G05 cell subpopulations, and state 4 mainly contained the G01 and G07 cell subpopulations (Fig. 5h), we further analyzed the difference in the percentage of G02 and G05 in state 3 and G01 and G07 in state 4 between the obese and normal groups. The results indicated that the percentage of G02 and G05 in state 3 of the obese group was significantly higher than that in the normal group (Fig. 5j). This suggests that G02 and G05 cell subpopulations are the primary components of the terminal transformation state in the developmental trajectory of granulosa cells, potentially playing a crucial role in regulating ovarian function decline under obese conditions.

Additionally, based on the pseudotime developmental trajectory, we compared the gene expression profiles between different cell fates (pre-branch: State 1; cell fate 1: State 3; cell fate 2: State 4) and performed pathway enrichment analysis for DEGs (Supplementary Fig. S2a). We observed significant upregulation of genes such as Fdx1, Gm42418, and Cyp11a1 in state 3, as well as upregulation of Ephx2, Fdx1, and Mrap in state 4 compared to state 1. Pathway analysis revealed enrichment in KEGG pathways related to steroid biosynthesis, terpenoid backbone biosynthesis, cortisol synthesis and secretion, and glutathione metabolism in state 3, while pathways related to terpenoid backbone biosynthesis, mineral absorption, ovarian steroidogenesis, and ferroptosis were enriched in state 4 (Supplementary Fig. S2b). In addition, we also compared the gene expression profiles and KEGG enrichment between G01 or G01&G07 in State04 and G02 or G02&G05 in State03 (Supplementary Fig. S3). These findings suggest that obesity alters the cell fate of normal granulosa cells, impairing their ability to regulate follicle maturation effectively.

### Obesity’s impact on intercellular communication between different cell types

**Fig. 6:**
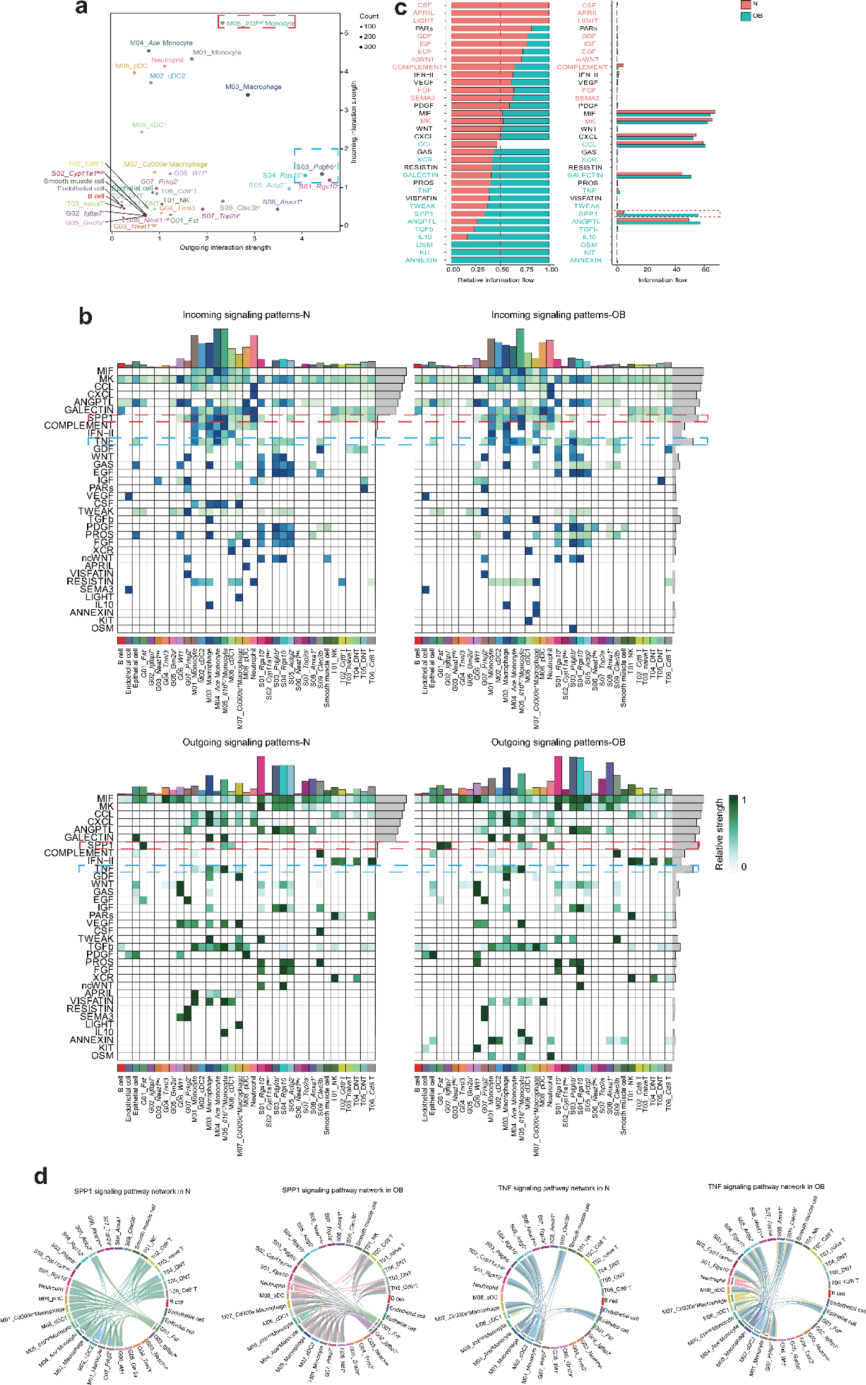
Analysis of intercellular communication between different types of cells. **a** Dot plot of the outgoing interaction strength and incoming interaction strength between different cell subsets from all ovarian cells. The blue and red dashed boxes indicate the strongest outgoing and incoming interaction strength of subsets of cells, respectively. **b** The incoming signaling patterns (upper part) and outgoing signaling patterns (lower part) between OB and N group. The red and blue dashed boxes indicate the SPP1 and TNF signals between OB and N group, respectively. **c** The relative abundance analysis of secreted cytokines between OB and N group. **d** SPP1 and TNF signaling pathway network for each cell sub-cluster from all cell types between OB group and normal group

Cell-cell interactions play a crucial role in ovarian fibrosis development. We explored how obesity affected intercellular communication among cells. After assessing intercellular interaction strength, we identified the outgoing interaction strength of S01 Rgs10^+^ and S03 Pdgfrb^+^ subpopulations of stromal cells as the strongest, while the incoming interaction strength of M05 Il1b^high^ monocytes was the highest (Fig. 6a). To delve deeper into the interactive relationships between cells, we defined outgoing and incoming signaling patterns (Fig. 6b). Notably, SPP1 levels significantly increased in the OB group compared to the normal group (Fig. 6b, c). Furthermore, SPP1 was predominantly secreted by G01 Fst^+^ subpopulations of granulosa cells in the normal group, but in the OB group, it was produced not only by G01 Fst^+^ subpopulations but also by G02 lgfbp7^+^ subpopulations (Fig. 6b, d). In incoming signaling patterns, the SPP1 signal was mainly received by M04 Ace^+^ monocytes and M05 Il1b^high^ monocytes in the normal group, whereas in the OB group, it was primarily received by M05 Il1b^high^ monocytes. In addition, we also found that TNF level mainly secreted by M05 Il1b^high^monocyte was upregulated in the OB group compared with the normal group. In the normal group, the reception of TNF signal was predominantly by the M04 Ace^+^ monocyte, while it was predominantly by the S03 Pdgfrb^+^ subpopulations of stroma cell in the OB group. Using CellPhoneDB, we defined the ligand-receptor relationship between them, and in conjunction with our differential analysis results, we identified two ligand-receptor pairs: SPP1-CD44 and TNF-TNFrsf1a (Supplementary Fig. S4a). The gene expressions of these pairs were influenced by obesity (Supplementary Fig. S4b). These results indicate a heightened interaction strength of SPP1-CD44 pairing within lgfbp7^+^ granulosa cell subtypes and Il1b^high^ monocyte subtypes in the ovarian tissues of obese mice. Moreover, the interaction strength between Il1b^high^ monocyte subtypes and Pdgfrb^+^ stromal cell subtypes in the form of TNF − TNFrsf1α interaction was also enhanced.

### Verification of the expression pattern of SPP1 and TNF-α

**Fig. 7:**
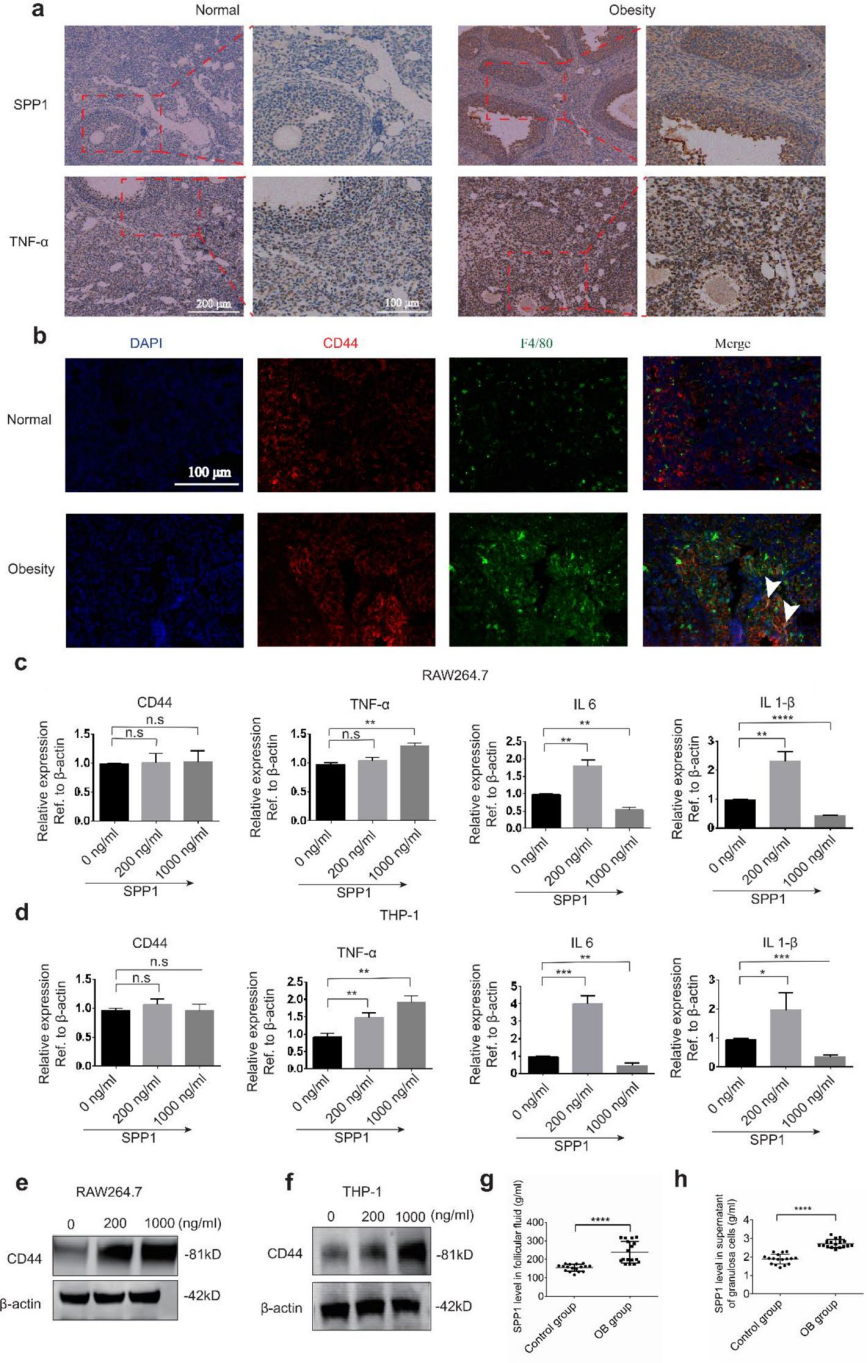
Verification of key genes expression pattern. **a** Immunohistochemical staining of SPP1 and TNF-α in ovarian tissue in the control group and the obese group. **b** Immunofluorescence staining of CD44 and macrophage marker F4/80 in ovarian tissue in the control group and the obese group. Arrows indicate cells expressing both F4/80 and CD44. Quantitative PCR analysis of the mRNA expression of CD44, TNF-α, IL 6 and IL-1β after treatment with indicated concentration of SPP1 recombinant protein for 24 h in RAW264.7 cell (**c**) or THP-1 cell (**d**). Western blot analysis of the expression of CD44 protein after treatment with indicated concentration of SPP1 recombinant protein for 24 h in RAW264.7 cell (**e**) or THP-1 cell (**f**). **g** Detection of SPP1 level in follicular fluid of obese and NW women. **h** Determine of SPP1 expression in granulosa cell culture supernatant of obese and NW women. (n.s, not significant; *P < 0.05; **P < 0.01; ***P < 0.001; ****P < 0.0001)

To validate our findings, we conducted immunohistochemistry to assess changes in the expression of key proteins in the ovaries. The results indicated a significant increase in the expression of SPP1 and TNF-α due to obesity (Fig. 7a). Importantly, SPP1 was primarily expressed in granulosa cells. Additionally, we used immunofluorescence to confirm that CD44, the receptor for SPP1, was predominantly expressed in macrophages (Fig. 7b). Interestingly, in vitro experiments demonstrated that treatment with SPP1 recombinant protein upregulated the expression of CD44 protein but not mRNA levels in mouse and human macrophage cell lines (Fig. 7e, f). Moreover, SPP1 treatment significantly increased the levels of proinflammatory cytokines, including TNF-α, IL6, and IL1-β (Fig. 7c, d). We further analyzed SPP1 levels in follicular fluid and granulosa cell culture supernatant from obese patients and normal-weight women, finding that SPP1 expression was significantly upregulated in obese patients compared to normal-weight women (Fig. 7g, h). These results suggest that the upregulation of SPP1 in granulosa cells induced by obesity promotes the inflammatory infiltration process in the ovary.

## Discussion

Obesity has a detrimental impact on the ovarian environment, leading to disruptions in reproductive hormone balance, lipid accumulation, inflammation, oxidative stress, insulin resistance, and mitochondrial dysfunction [16–19]. However, the exact mechanism by which obesity induces ovarian fibrosis remains unclear. Ovarian fibrosis is a complex phenomenon in mammals, necessitating the integration of diverse data types from various ovarian cell types for a comprehensive understanding of its regulation [20, 21]. In this study, we employ scRNA-seq techniques to explore the transcriptome dynamics of ovarian fibrosis in female mice after exposure to obesity. We meticulously examine the alterations in signaling communication pathways, specifically focusing on the developmental changes in granulosa cells due to obesity. Furthermore, we investigate how obesity affects interactions between various ovarian cell types.

Advancements in scRNA-seq techniques have enriched our ability to characterize ovarian cell populations with greater precision, enabling us to assign more accurate designations to individual cell types concerning their roles in both physiological and pathological processes [22, 23]. In this investigation, we identify distinct follicular niches, including granulosa cells, stromal cells, epithelial cells, endothelial cells, and immune cells. Our analysis reveals unique transcriptional profiles and cell communication patterns among granulosa cells and other cell types before and after exposure to obesity. These identified cell types and their gene expression changes during ovarian fibrosis offer valuable insights for future functional studies.

Previous studies on granulosa cells have primarily centered on their role in follicular development, often overlooking their contribution to ovarian fibrosis [24–26]. To comprehensively analyze the involvement of granulosa cells in obesity-mediated ovarian fibrosis, we employ various algorithms and bioinformatics analyses. By UMAP analysis of granulosa cells, we found that granulosa cells divided into seven cell clusters, with an increased percentage of G05 subclusters in the OB group. Surprisingly, the G05 subgroup significantly upregulates the expression of the CYP11A1 gene coding the cholesterol side chain cleavage enzyme which catalyzes the first step in the process of androgen synthesis, that is, the conversion of cholesterol to the precursor of androgen, pregnenolone, and is the key rate-limiting enzyme in androgen biosynthesis, which is also associated with polycystic ovarian syndrome (PCOS) induced female infertility characterized by hyperandrogenemia [27, 28]. We hypothesized that obesity leads to the upregulation of CYP11A1 in G05 cell subsets causing hyperandrogenemia, resulting in anovulation and infertility. Interestingly, we also observe that some granulosa cells exposed to obesity undergo a transformation in cell fate, resulting in a distinct cell state compared to normal granulosa cells. Through the examination of differentially expressed gene sets along this trajectory, we identify biological processes related to steroid biosynthesis, terpenoid backbone biosynthesis, cortisol synthesis and secretion, and glutathione metabolism. These findings suggest that obesity leads to an imbalance in reproductive hormone production within granulosa cells. Numerous existing studies have underscored the crucial role of immune cells in the development of ovarian fibrosis [29, 30]. In this study, we observe a deficiency of naïve T cells within the ovary following exposure to obesity, implying a functional imbalance compared to the normal group.

Throughout the course of ovarian fibrosis development, intercellular signaling communication among various cell types is pivotal for the activation of stromal fibroblasts, known for their role in collagen secretion [31]. To investigate the intercellular communication among different cell types and to elucidate the impact of obesity on the ovarian microenvironment, we map the secretory signaling network within the ovary. Our analysis identifies ovarian stromal cells and monocytes as primary contributors to outgoing and incoming secretory signaling, respectively. Notably, granulosa cells predominantly upregulate the secretory signal SPP1, shedding new light on their role in the ovarian microenvironment. SPP1 is a multifunctional glycoprotein initially recognized as a proinflammatory cytokine secreted by T cells and later found in various tissue-resident macrophages, associated with apoptotic cell clearance, chemotaxis, and macrophage migration [32, 33]. Schepper et al. found that SPP1 protein, as an exogenous signal, regulates synaptic phagocytosis through paravascular transforming growth factor-1β and receptor signals on microglia [34].

Our study reveals the existence of a subpopulation of ovarian granulosa cells known as ovarian lgfbp7^+^ granulosa cells. Under normal conditions, these cells exhibit minimal SPP1 expression but upregulate it in response to obesity. The primary recipients of SPP1 are ll1b^high^ monocytes. In vitro experiments confirm that exogenous SPP1 induces the upregulation of TNF-α, IL-6, and IL-1β expression in macrophages. These results suggest that lgfbp7^+^ granulosa cells can activate macrophages through SPP1 secretion. Macrophages play various essential roles in regulating the ovarian microenvironment, synthesizing diverse cytokines, and engaging in crosstalk with granulosa cells and stromal cells. Traditionally, it was believed that obesity and aging were associated with an increased presence of inflammatory (iNOS^+^) M1 macrophages and anti-inflammatory (CD163^+^) M2 macrophages, both known to stimulate fibrotic collagen deposition by stromal fibroblasts [35, 36]. Our observations indicate that ll1b^high^ monocytes, when elevated, increase TNF-α expression, primarily received by S03 Pdgfrb^+^ subpopulations of stromal cells. These findings suggest that macrophage activation of stromal cells through TNF-α upregulation contributes to the fibrotic collagen secretion. In summary, we propose that obesity disrupts the developmental trajectory of granulosa cells, resulting in increased secretion of SPP1, which is necessary for macrophages to upregulate inflammatory markers TNF-α, ultimately stimulating stromal fibroblasts to secrete fibrotic collagen, thereby impairing ovulation.

## Conclusions

Collectively, our findings provide fresh insights into the mechanisms underlying premature ovarian dysfunction in premenopausal women with obesity. We elucidate the interacting and inflammatory stress pathways driving fibrotic collagen deposition, which leads to impaired ovulation. Now, subfertility caused by obesity can be improved through lifestyle interventions and/or drug treatment [37]. Drugs, including metformin, liraglutide, and clomiphene can be used to increase the probability of conception. We postulate that these processes may be amenable to reversal through pharmacological interventions, for example, reducing the expression level of SPP1 in the G05 cell subpopulation, potentially offering an avenue for enhancing fertility and extending normal ovarian function in obese women.

### Electronic supplementary material

Below is the link to the electronic supplementary material.

Supplementary Material 1

## Data Availability

No datasets were generated or analysed during the current study.

## Abbreviations

scRNA-seq: Single-cell RNA sequencing
OB: Obesity
BMI: Body mass index
NW: Normal-weight
IVF: In vitro fertilization
DEGs: Differentially expressed genes
GO: Gene Ontology
KEGG: Kyoto Encyclopedia of Genes and Genome
PFA: Paraformaldehyde
RT-qPCR: Reverse transcription quantitative polymerase chain reaction
ELISA: Enzyme-linked immunosorbent assay
AMH: Anti-Müllerian hormone
FSH: Follicle-stimulating hormone
E2: Estradiol
SPP1: Secreted phosphoprotein 1
ANOVA: Analysis of variance
HFD: High-fat diet
HE: Hematoxylin-eosin
UMAP: Uniform Manifold Approximation and Projection
