# Supplementary material for: Single-cell transcriptomic profiling unveils insights into ovarian fibrosis in obese mice
Source: Biol Direct. 2024 Jul 2;19:52. doi: 10.1186/s13062-024-00496-9 (PMC11218254; doi:10.1186/s13062-024-00496-9)
Supplement: Supplementary file 1 — Supplementary Material 1 [file 13062_2024_496_MOESM1_ESM.docx]

**Figure S1**


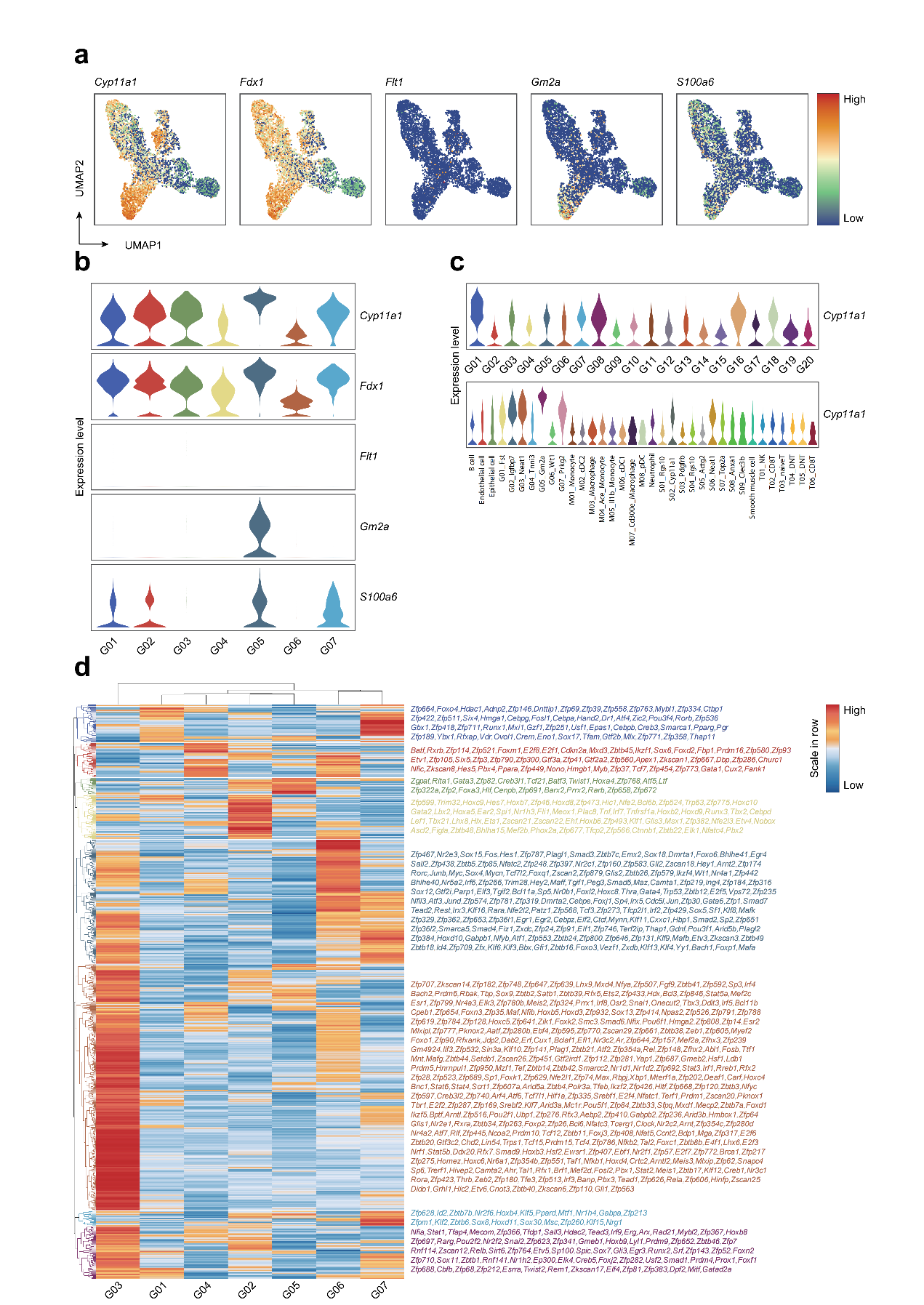


**Figure S2**


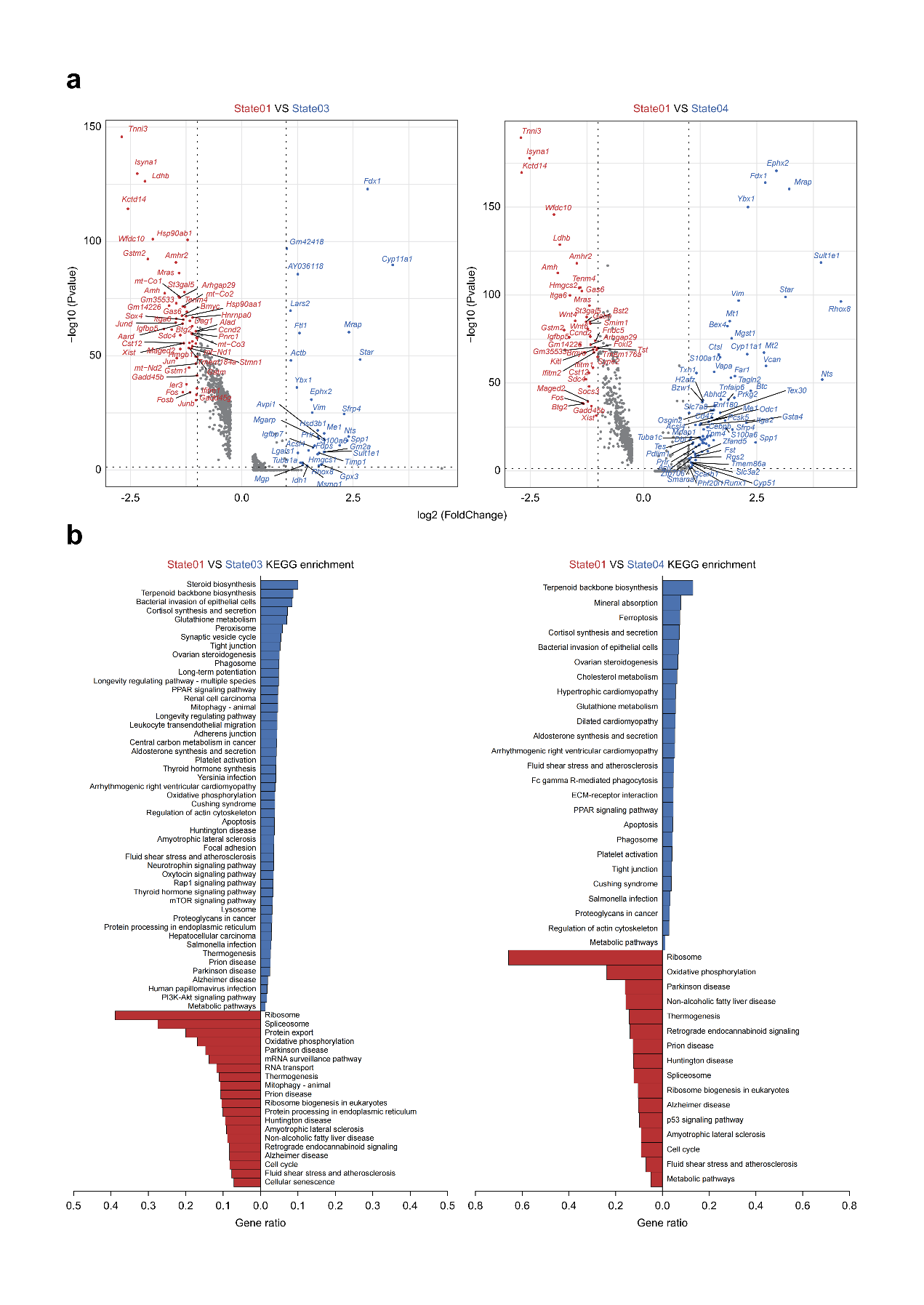


**Figure S3**


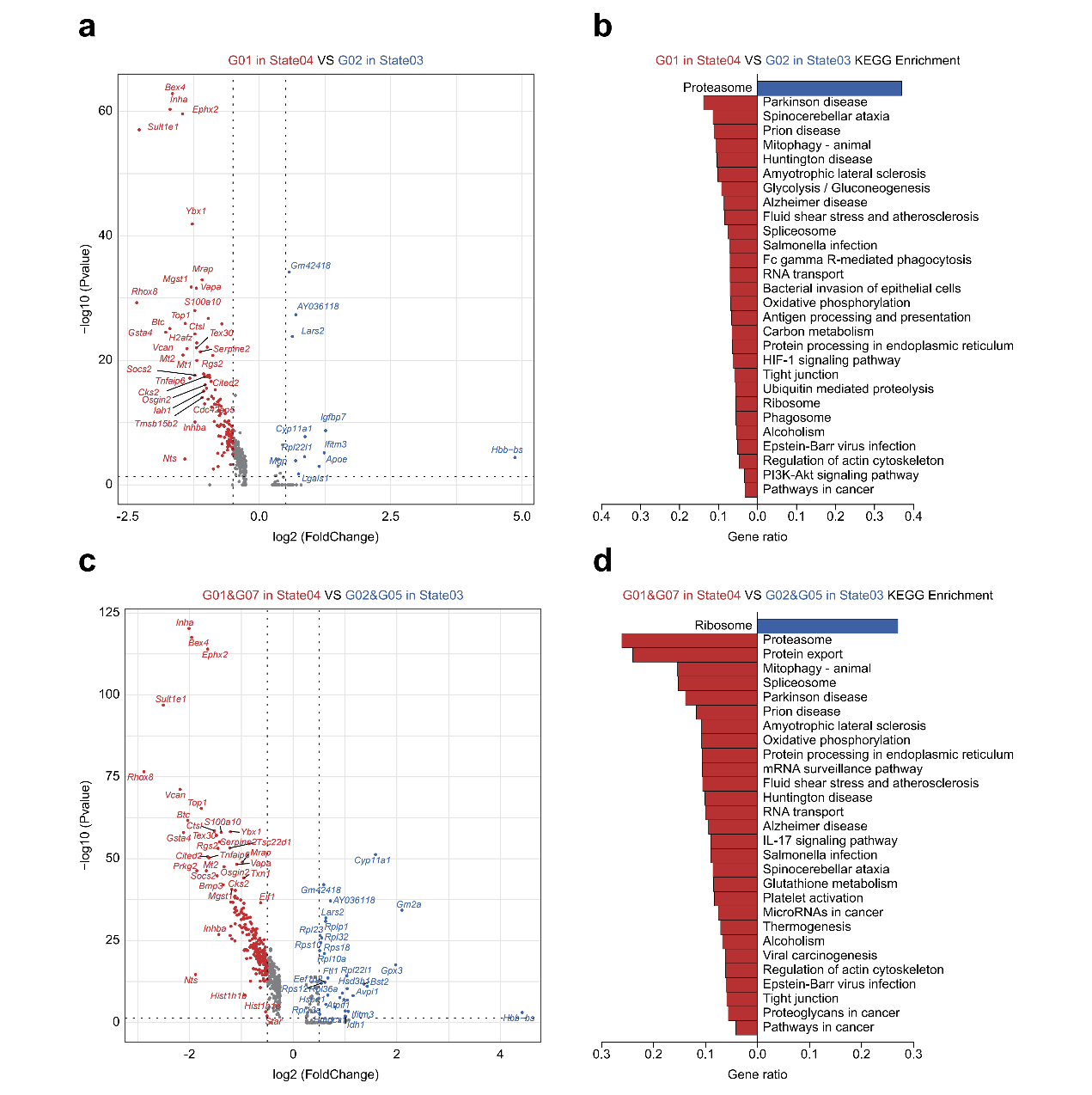


**Figure S4**


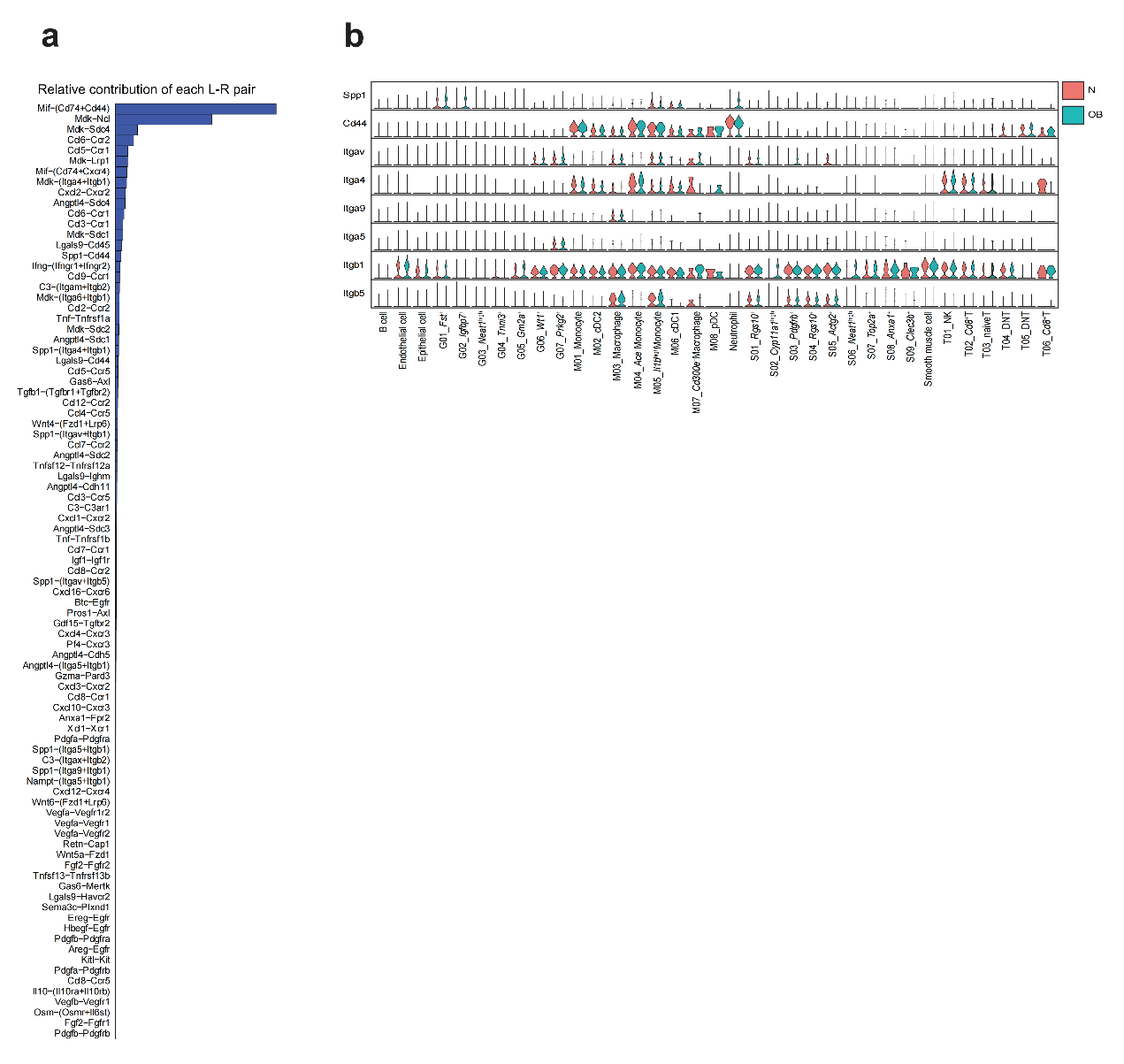


| **Table S1. Participant Characteristics and Serum Concentrations** | | | |
| --- | --- | --- | --- |
|  | BMI 18.5-24.9 kg/m2 (N=17) | BMI＞25 kg/m2 (N=12) | *P* value |
| Age, y | 32.5±0.4 | 31.9±1.3 | 0.0834 |
| BMI, kg/m2 | 20.8±1.2 | 30.6±2.9 | < 0.0001 |
| Pregnancy, N | 14 | 8 | 0.4029 |
| Live births, N | 11 | 6 | 0.4713 |
| Male factor, N | 4 | 2 | 1.000 |
| Endometriosis, N | 3 | 2 | 0.9450 |
| Ovulatory disorder, N | 5 | 3 | 0.7935 |
| Tubal factor, N | 3 | 3 | 0.6302 |
| Unexplained, N | 2 | 2 | 0.7061 |
| AMH, ng/mL | 2.920 ± 0.4807 | 4.102 ± 0.8959 | 0.2490 |
| FSH,mIU/mL | 8.846 ± 2.078 | 5.818 ± 0.4589 | 0.4344 |
| E2, pmol/L | 288.5 ± 80.62 | 110.5 ± 13.36 | 0.2408 |
| Progestin, nmol/L | 2.972 ± 1.867 | 1.326 ± 0.9333 | 0.6372 |
| Prolactin, mIU/L | 540.6 ± 159.9 | 287.1 ± 23.61 | 0.3659 |
| LH, mIU/mL | 10.80 ± 2.899 | 2.394 ± 0.1434 | 0.1279 |
| Testosterone, nmol/L | 3.076 ± 1.905 | 0.7800 ± 0.1108 | 0.4741 |
| Glucose, mg/dL | 103.6 ± 2.7 | 104.5 ± 1.1 | 0.2862 |
| LDL, mg/dL | 68.3 ± 10.2 | 73.6 ± 8.6 | 0.1539 |
| HDL, mg/dL | 44.9 ± 6.1 | 40.4 ± 5.9 | 0.0576 |
| Total cholesterol, mg/dL | 226.9 ± 10.5 | 230.7± 4.6 | 0.2514 |
| C-reactive protein, mg/mL | 3.4 ± 0.4 | 4.1 ± 1.6 | 0.0932 |

**Table S2. Primer Sequences used for RT-qPCR**

| Genes | Forward primer | Reverse primer |
| --- | --- | --- |
| Mouse CD44 | 5′-CAAGTGCGAACCAGGACAGTG-3′ | 5′- AATCAGAGCCAGTGCCAGGAG-3′ |
| Mouse TNF-α | 5′-AAGACACCATGAGCACAGAAAGC-3′ | 5′- GCCACAAGCAGGAATGAGAAGAG-3′ |
| Mouse IL 6 | 5'-GAAACCGCTATGAAGTTCCTCTCTG-3' | 5'- GTATCCTCTGTGAAGTCTCCTCTCC-3' |
| Mouse IL 1-β | 5'-CACCTCACAAGCAGAGCACAAG-3' | 5'-GCATTAGAAACAGTCCAGCCCATAC-3' |
| Mouse β-actin | 5'-TATCGCTGCGCTGGTCG-3' | 5'-CCCACGATGGAGGGGAATAC-3' |
| Human CD44 | 5'-CTGCCGCTTTGCAGGTGTA-3' | 5'-CATTGTGGGCAAGGTGCTATT-3' |
| Human TNF-α | 5'-CCTCTCTCTAATCAGCCCTCTG-3' | 5'-GAGGACCTGGGAGTAGATGAG-3' |
| Human IL 6 | 5'-ACTCACCTCTTCAGAACGAATTG-3' | 5'-CCATCTTTGGAAGGTTCAGGTTG-3' |
| Human IL 1-β | 5'-AGCTACGAATCTCCGACCAC-3' | 5'-CGTTATCCCATGTGTCGAAGAA-3' |
| Human β-actin | 5'-CATGTACGTTGCTATCCAGGC-3' | 5'-CTCCTTAATGTCACGCACGAT-3' |
